# When a Doctor Becomes a Patient with a Mystery Illness: A Case Report

**DOI:** 10.1155/2010/565980

**Published:** 2010-07-04

**Authors:** Brit Haver

**Affiliations:** Department of Clinical Medicine, Section for Psychiatry, University of Bergen, PB 23, Sandviken, 5812 Bergen, Norway

## Abstract

Symptoms presenting as mental disorders may represent epileptic discharges, especially from the temporal lobe. Both mental and somatic symptoms are common in temporal lobe epilepsy, which may confuse doctors, leading to extensive medical examinations and tests, false diagnoses, and ineffective treatment. Also, the episodic nature and variety of symptoms between as well as in individual cases hinder correct diagnosis. Since epileptic discharges may be visible on EEG only during an epileptic fit—and may need highly specialized equipment to detect—many cases are undiagnosed or treated under false diagnoses. The author believes that undetected temporal lobe epilepsy falsely labelled as psychiatric disorders are common. Specific and effective treatment exists for temporal lobe epilepsy, making correct diagnosis important. This history—based on the author's personal experience—also illustrates aspects of the physician-patients' problems and resources, as well as the gap between somatic and psychiatric medicine concerning this rather common neuropsychiatric disorder.

## 1. Introduction

Being a doctor might represent a specific stress situation when you enter a hospital as a patient—and also for the team of doctors providing the diagnosis and treatment [1, 2]. Being a psychiatrist and a patient at a somatic hospital may represent an extra challenge, especially if you show symptoms of mental instability. Disorders perceived as “mental” often are of little interest for somatic doctors, and the competence of somatic doctors to diagnose mental disorders may be limited to a collective term for those conditions where no “organic” causes can be found. Psychiatrists on the other hand may be regarded by their somatic colleagues to have little competence in disorders of the body. Of special relevance is the “iron curtain” seemingly dividing the worlds of neurologists and psychiatrists [3], areas of medicine dealing with the same organ systems, which should include body and mind. In the following I will present a case history illustrating these phenomena in a real life situation. My question is: did my symptoms and behaviour simply match the somatic doctors' general ideas of psychiatrists, namely a group of doctors with an odd character?

## 2. Case Presentation

Some years ago, I happened to be a patient at a university hospital. None of the doctors could find any explanation for my symptoms, which suddenly made me unable to work and live an independent life. Since my body had been screened thoroughly—including MRI of the brain, EEG and an endless list of lab tests—without finding anything wrong—I was given up and labelled a psychiatric case. My symptoms must be related to stress, depression, burn-out or similar diffuse processes like “meeting the wall”, I was told. As a psychiatrist myself, I knew that no psychiatric condition could explain my symptoms. But, by then the somatic doctors—including gastroenterologists, endocrinologists and general internists seemingly had lost their interest in me. They simply stopped listening with an open mind, and said there was nothing more they could do for me. I felt terrible.

It started with gastrointestinal and upper respiratory symptoms during a short stay abroad. I had an episode feeling “strange” during that stay, and another episode during the flight back. However, these episodes passed so quickly that I did not think they were of any importance. Soon afterwards, one morning when going to work, I found my purse left outside the house, the car and garage door open, all groceries from the car strewn disorderly in the driveway, and even the front door open. I was unable to memorize what had happened after returning from work the day before. Immediately I saw a doctor and was admitted to the hospital. Appropriate tests were performed, focusing on the gastrointestinal tract, the brain and identifying the infectious agent. I left the hospital with a diagnosis of unspecified gastroenteritis and felt fine. Another time, I woke up during the night feeling “dead”: unable to move, I heard no heart beats and felt no respiration movements. However, since I was able to think, I realized I could not be all dead. So, while thinking: “What can this be?” I felt as if coming up from deep under the sea. Suddenly I was awake and could move. I thought maybe I had had an episode of hypoglycaemia and found something to eat. At least something was terribly wrong, and I was admitted to the hospital a second time. Now, my gastrointestinal symptoms were gone, and the doctors focused on endocrinology and the brain. Again, no definite diagnosis was established, while one of the doctors said he thought my condition was due to a depression. When I asked him to mention some criteria for the diagnosis of depression, he responded: “Well, as a psychiatrist you should know, shouldn't you?” 

In the following, my symptoms were fluctuating, episodic and changing. In between, I could feel fairly well and thought that even if I did not understand what was going on, everything would be all right as time went on. However, by time, my situation worsened: vegetative symptoms like feeling hot or cold, blushing, nausea, fluctuating blood pressure as well as miosis of the pupils were observed. Also, temporary cognitive symptoms like slow speech, difficulties finding ordinary words, and a peculiar feeling that my thoughts were caught up, repeating themselves endlessly for a period of time. These times I felt a different person than my ordinary self, which was very frightening. Also, my mood was swinging between extremes: being “high” and talkative for a day or two, and very depressed and withdrawn the next. I had never had similar experiences.

After months of repeated hospital admissions, I felt exhausted and desperate. I had the impression that the doctors as a group had concluded that my condition was a mental disorder. At last, I refused to leave the hospital until an explanation and a possible treatment had been found. Since I believed that I could not be the first person to acquire a syndrome like this, I resorted to my old medical textbooks. The condition must have been described and named by someone. Based on what I found, I made a list of 12 possible causes and presented the list for the doctors. One of the disorders on my list was epilepsy. The episodic nature of my symptoms was a strong indicator of such a diagnosis. But none of the doctors showed any interest in my suggestions.

Luckily, during that last hospital stay I had a fit of symptoms on my way to the EEG lab. This time, flicker stimulation was extremely painful: it felt like an electric current went through my head. I screamed, and the procedure had to be interrupted. A neurologist was called who told me that my pain was due to anxiety. So I said to him: “If it is anxiety, doctor, then maybe it would help me if you hold my hand?” The doctor approached me, hesitating. His hand was clammy, maybe because he had been warned about me being a very difficult patient. But flicker stimulation again had to be stopped. A purportedly calming hand did not change my reaction.

This time the EEG was pathological: there were spikes in the left fronto-temporal region. At last, a diagnosis of epilepsy had been established. By that time, I was close to giving up on life, since the fits kept coming more frequently and lasted longer. I was frightened to stay alone in my house, especially at night. My weight had dropped twenty pounds, and I was starting to look like a cancer patient. The day the treatment was initiated, a condition similar to a morbus Addison was disclosed. My adrenal glands were about to give up as well.

## 3. Discussion

Based on my history, one might ask if women are more easily diagnosed with “functional” or mental disorders when doctors do not find an organic disorder explaining their symptoms. My feeling is that this still is the case, as “Hysteria” mostly was a female condition. Another issue is the problem of being a doctor undergoing medical examinations by other doctors who might feel intimidated by your professional knowledge. What impact does that make on the diagnostic process and the treatment obtained? In this specific case, did my symptoms and behaviour simply match the somatic doctors' general ideas of psychiatrists, namely a group of “mad” doctors with an odd character or a low level of medical knowledge—or both? In the aftermath, I have wondered: how could it go that far? Usually, doctors are warned not to diagnose nor treat themselves. In my case, I believe that being a doctor simply saved me. Few months after the treatment had started, my adrenal glands were well-functioning, I gained weight and gradually went back to my premorbid way of life. So, these symptoms related to my general condition were secondary to the primary disorder of temporal lobe epilepsy. The epilepsy might have been precipitated by an unspecified infectious disorder during a stressful situation abroad.

But why tell this story years after it happened? My answer is that doctors in all medical fields have something to learn from this case history. Lack of general medical knowledge overriding our own narrow field of specialty may result in many false diagnoses, wherever you work in medical practice. Overly beliefs in lab tests and underestimation of the value of skilled observation of clinical symptoms as well as patient interviews, are common sources of errors in medicine generally today. These attitudes may conceal the doctor's insecurity and feeling of helplessness when confronted with patients who do not conform to a simple diagnostic category, resulting in false or missed diagnoses. The cognitive psychology of missed diagnoses has been excellently described [4, 5]. The pitfalls include relying on initial impression, a failure to consider additional diagnoses after an initial diagnosis, premature closure or blind obedience to authority or technology. Another source of error is the lack of checking for disconfirming evidence. In the present case, what are the chances that a middle aged woman suddenly starts a career as a revolving door patient at a somatic hospital, due to anxiety or depression? Also, a missed diagnosis could be due to the framing effect: for example in the present case: what information did the referring doctor provide at first admission? Could this information have led the doctors to a premature closure instead of revising my case in a frame of more complete information? In the aftermath, I learned that the referring doctor had diagnosed and presented my case as a “mental disorder”.

Another aspect of this case history is that in the end, two separate disorders were diagnosed. Physicians typically expect only one explanation for a host of symptoms, and may incorrectly use symptoms of one disorder to limit the consideration of a second disorder. Also, intermittent and changing symptoms are a challenge to the clinician, since they may have disappeared when the patient comes to the examination. Fluctuating symptoms are more likely due to epilepsy, but may occur in vascular, metabolic, autoimmune and endocrine disorders.

Again, why tell this story? Another answer is that I believe that fluctuating and episodic psychiatric symptoms often may indicate a neuropsychiatric condition due to epilepsy. Typically, these patients are referred to neurological departments—or directly to psychiatric institutions. Because of the wide spectre of symptoms they may however enter a variety of sections within general medicine—or even vacillate between certain subspecialties without a correct diagnosis. Third, since these conditions are both very painful and frequently misdiagnosed, they often result in major disability or even suicide. Fourth: because very effective treatment exists. Among all epileptic conditions, temporal lobe epilepsy is probably the most frequently undiagnosed condition—or obtaining a delayed diagnosis—since its symptoms can mimic a wide variety of somatic and psychiatric disorders, without showing any of the classical symptoms of epilepsy such as grand mal seizures or loss of conscience [6–8].

My general advice for the somatic and psychiatric doctors follows:

1. When you do not find a specific somatic disorder, this does not mean that the disease is “mental”, even if the patient has mental symptoms. Any serious somatic disorder may precipitate symptoms related to stress, anxiety and depression.
2. Patients with episodic symptoms should ideally be investigated and diagnosed when under a fit of symptoms. This may be essential for epilepsy as well as other disorders with intermittent symptoms. Also, the location of electrodes may be critical for the diagnosis of temporal lobe epilepsy [9]. An ordinary scalp EEG may be negative, while nasopharyngeal electrodes may show inter-ictal spikes [9]. Prolonged video EEG monitoring and studies under sleep deprivation may be necessary.
3. When you do not understand patients with complex clinical symptoms, take a new, comprehensive history from your patient—before you order new tests or prescribe another ineffective medication. Make a time-line between the start and the progression and course of illness. Start with an open mind, including findings in the medical record leading in a certain direction—or excluding certain syndromes. What are the patient's beliefs about the disorder? “Trial and error” tests are usually of little help in this situation, since they cost a lot, mislead the doctors by producing false or irrelevant “positive” result, thereby postponing the correct diagnosis. In epilepsy, a detailed history leads to an accurate diagnosis in 90% of the cases [10].
4. Epilepsy is frequently under-recognized in patients experiencing abrupt changes in psychiatric symptoms and behaviour. The relationship between mood disorders and epilepsy is thought to be bidirectional [11, 12], and antecedent suicide attempt is a major risk factor [13]. Subclinical seizures might masquerade as non-ictal depression [14].
5. Temporal lobe epilepsy could start at any time during the life cycle and also complicates the clinical picture of patients with serious psychiatric disorders [15]. Some of these patients have had febrile seizures as a child or have a family history of either epilepsy or febrile seizures, or both [16].
6. Partial seizures could also present as ictal anxiety, mimicking panic attacks [17]. In my case, anxiety disorder was the most frequently discussed diagnosis. Mechanisms regulating neuronal excitability in amygdala have recently been reviewed, explaining the close relationship between stress, anxiety disorders and the pathogenesis of temporal lobe epilepsy [18].
7. The complex relationship between psychopathology as presenting feature of epileptic seizures, psychiatric comorbidity of epilepsy as well as interictal psychiatric diagnoses specific to epilepsy have been distinguished recently [19].

In my case, treatment with clobazam (Frisium) was very effective preventing new fits. However, it took about a year until my condition stabilized, allowing a return to my teaching job at the university. Today, I am well without medication. Some memory problems still exist, though, which I believe are related to long term “firing” by a few pathological cells in my left temporal lobe during those very painful days [20].

## 4. Conclusion

Temporal lobe epilepsy may mimick or complicate a variety of psychiatric and somatic disorders, leading to major disability, a number of false diagnoses and ineffective treatment regimens. A comprehensive case history indicating a complex list of fluctuating symptoms may lead to a correct clinical diagnosis, verified by use of specialized EEG equipment, including prolonged video EEG monitoring. Effective treatments exist, making it even more important to think about this not uncommon condition.
